# The aftermarket performance of initial public offerings: New evidence from an emerging market

**DOI:** 10.1371/journal.pone.0272092

**Published:** 2022-08-22

**Authors:** Dilesha Nawadali Rathnayake, Zhixin Zhang, Bai Yang, Pierre Axel Louembé

**Affiliations:** 1 School of Economics, Shandong University of Technology, Zibo, PR of China; 2 Business Achool, Shandong University of Technology, Zibo, PR of China; 3 School of Accounting, Dongbei University of Finance & Economics, Dalian, PR of China; UNITED STATES

## Abstract

This paper presents new updated evidence on the initial public offering (IPO) aftermarket performance for 144 public listed firms on the Colombo Stock Exchange from 1991 to 2017. We found that average aftermarket returns are always lower than 1%. On average, buy and hold abnormal returns are negative in a short period, and abnormal returns gradually become positive over a longer period (12.46% in 3 years). Further, aftermarket returns are positively related to investor sentiment and the annual volume of listings while being negatively related to initial returns, which is consistent with the divergence of opinion hypothesis. We suggest that investors should hold their subscriptions of IPO shares for a prolonged time, usually exceeding two years, as the dynamic of shares rewards the investors with positive abnormal returns in the long run.

## Introduction

Share trading has been a part of Sri Lanka’s history since 1896, but it is only a few years later that an official Stock exchange was created, herein Colombo Stock Exchange (CSE) which has remained the main Stock market in the country. CSE is endowed with a fully automated trading platform and 20 business sectors listed. CSE witnessed an unprecedented expansion in 2009 to become the world’s best performing market of 2010, with a growth of 111.14%. Mainly due to the sound political improvements instigated since 2009 and a peaceful environment after the post-war period, CSE has considerably evolved and deserved additional attention from scholars and practitioners of finance. In fact, with a market capitalization of USD 16.07 billion in December 2018 and 202 IPOs launched between 1991 and 2017, the performance of such a developing market is worth being assessed.

Although the literature on IPO performance is rapidly growing, still there is a variety of results due to the different academic viewpoints applied, selection of determinants, measurement of performance and the contextual nature of individual firms. Despite the rather abundant empirical literature on IPO performance, the previous studies lead to a broad, diverse and multilateral set of findings due to the theoretical perspectives being adopted (determinants, performance measures, contextual nature of individual firms). Moreover, institutional, legal frameworks in emerging economies are not advanced compared to developed nations. Also, most research stressing the IPO performance are conducted in developed economies and large stock markets when emerging economies show substantial differences regarding economic growth, business environments, income levels, and management practices. Only a few studies evaluate the behavior of IPOs in developing nations such as Sri Lanka, which operate in challenging environments (civil war, political instabilities, Asian crisis, Tsunami devastation) and so far, manage to perform strongly in the corporate sector. Especially based on the challenging economic and political atmosphere where Sri Lankan companies perform comparatively strongly, research on the capital market is expected to give exciting outcomes and fill the existing gap in knowledge of the association between IPO performance in the long run.

Initial Public Offering (IPO) aftermarket performance is broadly documented and has been a subject of attention among scholars for decades [1,2]. Peter [3] investigated aftermarket returns of Sri Lankan IPOs in terms of privatized and non-privatized offerings using the market-adjusted buy and hold returns (BHRs). IPOs are initially underpriced, and those excess returns tend to decline by the end of three years. In contrast, privatized IPOs contribute higher returns than non-privatized IPOs in the Colombo Stock Exchange (CSE). The number of IPOs examined by Peter [3] was relatively small. Ediriwickrama and Azeez [4] studied aftermarket IPO underperformance in the CSE with calendar time techniques from 2000 to 2012 and identified several factor models to describe the return variation of IPO stocks in CSE. None of the studies have considered the determinants of aftermarket performance. This is the first study considering the wider time span and twelve determinants of IPO aftermarket performance in Sri Lanka.

This study presents new findings on IPO aftermarket performance for 144 Sri Lankan IPOs that went public from January 1991 to December 2017. We measured the IPO aftermarket performance up to 36 trading months (720 trading days), including the listing day returns. Further, this study focuses on the importance of IPO issue characteristics at the time of going public to find interpretations for the IPO aftermarket performance. The key goal of this research paper is to present updated evidence by examining the amount of IPO aftermarket returns in CSE, focusing on the market-adjusted abnormal returns. This study contributes to the IPO literature by presenting new findings of IPO aftermarket performance in the CSE, using an inclusive sample and a complete analysis of IPO returns. Thus, we carry out critical analysis to determine whether our results about the IPO aftermarket performances in Sri Lanka are similar to those found in previous literature for other emerging countries. Generally, there are three ways in which the study contributes to the current literature. First, the most recent dataset is considered to uncover aftermarket performance. Previous studies have covered a shorter period and smaller samples. Second, both market-adjusted cumulative average returns (CAARs) and average market-adjusted BHRs have been employed in this study to assess the aftermarket performance of IPOs. The outcomes deliver significant information and understanding for stakeholders to invest in IPOs. Based on our results, we recommend that stakeholders should be careful while analyzing IPO returns in the long run.

This paper is ordered into six headings. Section 2 explains the literature review, and section 3 summarizes the data and research methodology. Section 4 includes the empirical results and analysis. Last, section 5 presents the conclusions of the research.

## Literature review

The existing studies have presented numerous explanations for the behaviour of IPO aftermarket performance. However, there is a lack of observable variables that can describe aftermarket performance. To explore the determining factors of IPO aftermarket performance, several theories are considered in this study.

The divergence of opinion hypothesis suggests that the uncertainty about an IPO can attract overvaluation on a listing day, followed by underperformance in the long run. Miller [5] proposed that at first, investors lean towards being over-optimistic about the IPO value, which causes initial under-pricing and that later, as the differences of opinions reduce when information flows increase with time, the price of IPOs diminishes to the intrinsic value, producing low aftermarket performance. Gao et al. [6] provided further evidence for Miller’s [5] argument. The study which is based on 4,057 IPOs found that divergence of opinion, proxied by short-term stock return volatility (first 25 trading days after issuance), is negatively related to IPO long-term abnormal returns. In addition, the authors highlight the effect of market regulatory settings on assets early pricing. That is, the regulatory induced pricing bias and short-selling constraints could lead to inflated initial aftermarket IPO prices that autocorrect in the long run, resulting in aftermarket underperformance. As Short-selling is typically forbidden in CSM, investment opinion divergence, proxied by market volatility (first 40 trading days after IPO) throughout this investigation, shall also bear the negative sign reported in previous works. Following previous studies [6,7], ex-ante uncertainty is used as a proxy to analyze the relationship between the divergence of opinion and IPO aftermarket performance in CSE. Greater values of ex-ante uncertainty indicate a greater divergence of opinion for the IPOs. As such, the hypothesis predicts a positive relationship between the ex-ante uncertainty and the aftermarket performance.

The impresario hypothesis asserts that the IPO market is exposed to manipulations due to the presence of the investment banks, which are comparable to the ‘impresarios’ that would voluntarily under-price the new shares with the aim of attracting more investors to the securities’ markets Shiller [8]. Interestingly, this hypothesis points out the reliance on underwriters for certifying the quality of the new issue. Similarly, the impresario hypothesis is in line with the overreaction hypothesis [9]. The deliberate under-pricing of shares generates the appearance of an excess demand, which triggers investors’ optimism and channels an overreaction toward the stock. The misevaluation of shares in initial IPO markets will autocorrect over the medium run and the long run when extra information becomes accessible to the general public [10]. Both hypotheses predict IPO aftermarket performance to be negatively associated with the initial under-pricing. Conversely, signalling theory suggests that IPO under-pricing is positively related to IPO aftermarket performance in the long run [11]. During hot issue periods, high quality firms will issue IPOs and under-price the IPO shares to pass the signal of good quality to win the confidence of investors [12]. Loughran and Ritter [1] and Ritter [2] claimed that a firm that goes public in a hot issue period usually generates a high return in the short run and low returns in the long run.

Many studies have suggested that the initial returns and aftermarket performance have a negative association, in line with the overreaction hypothesis [2,8,13]. Further, Ritter [2] found that IPO aftermarket underperformance usually continues up to 3–5 years after listing. Nevertheless, the degree of IPO aftermarket underperformance is associated with whether the IPOs are either underpriced or overpriced on the first trading day. If the IPOs are underpriced on the first trading day, then initial returns would either not be related or be positively related to IPO aftermarket performance. However, if IPOs are overpriced on the initial trading day, then the initial returns will be negatively related to aftermarket performance because the initial overpricing will be corrected gradually by the post-IPO market. Subsequently, it is expected that IPOs with greater under-pricing will perform worse in the long run [8]. Following Chi and Padgett [14] and Rathnayake et al. [15] the raw initial returns (IR) and the market-adjusted abnormal return (MAAR) for each IPO on the first day of trading are calculated as Eqs 1 and 2, respectively.

$$IR_{i1}=\left[\left(P_{i1}-P_{i0}\right)/\left(P_{io}\right)\right]$$ (1)

$$MAAR_{i1}=\left\{\left[\left(1+IR_{i1}\right)/\left(1+Rm_{1}\right)\right]-1\right\}$$ (2)

Where *P*_i1_ = the closing price on the first trading day and *P*_*i0*_ = the IPO offer price of the *i*^th^ stock; *IR*_*i1*_ = the initial returns of the first trading day; *Rm*_*1*_ = the first trading day market return using the *Rm*_*1*_ *= [(Rm*_*1*_ *-Rm*_*0*_*)/ Rm*_*0*_*]* formula; *Rm*_*1*_ = the closing market index value on the first trading day of the *i*^th^ stock; and *Rm*_*0*_ = the closing market index value on the offering day of the stock.

Older firms perform better than are younger firms, as young firms generally have more ex-ante risk than do mature firms and mature firms have less information asymmetry with investors [2,16,17]. Thus, a positive relationship between firm age and aftermarket performance is expected. However, Brau, Couch, and Sutton [18] reported an insignificant negative relationship between issuer age and the long-term performance of IPOs. Belghitar and Dixon [16] and Ritter [2] documented a positive relationship between firm size and IPO aftermarket performance, as have other researchers who have used offer size as a proxy for ex-ante uncertainty [19–22]. Based on the divergence of opinion we expect a positive relationship between the offer size and aftermarket performance of IPOs. Board type is included in the study as a dummy variable, and we expect a positive relationship between Main Board listed companies and IPO long-term performance. Ritter [2], in the USA, and Levis [23], in the UK, for Main Board listings, found that IPOs with small size issues perform poorly in the long run. Following previous evidence, a positive coefficient is expected for Main Board listings in CSE. Following Thomadakis, Nounis and Gounopoulos [22], who found a significant negative relationship between IPO offer price and long-term market performance, we expected IPO price to be negatively related to aftermarket performance. Recently, Bhabra and Pettway [17] found a significant negative relationship, whereas while Mumtaz and Ahmed [24] found a positive but insignificant relationship, between long-term stock returns and the market volatility variable. We have computed market volatility as “the standard deviation of daily market returns over the first 40 trading days after the closing date of subscription” [25], and a negative relationship is expected.

We have included the hot dummy variable, which takes the value of 1 for the hot years, and 0 otherwise, to differentiate between hot and cold IPOs. Following the windows of opportunity hypothesis, a negative post-issue IPO performance [2,26] is expected. However, a positive relationship between the IPO volume in the market and aftermarket performance has been found by several prior studies [27,28].

Investor’s sentiment is found to be positively correlated to the IPO performance on the first trading day, and the observed share subsequently underperforms over the long run [10,29]. However, Khan, Ramakrishnan, Haq, Ahmad, and Alim [30], and Dimovski and Brooks [31] illustrated a significant positive relationship between market sentiment and aftermarket returns in a sample of Malaysian firms. Therefore, we predict that sentiment and aftermarket performance are negatively related. Nevertheless, Perotti and Oijen [32], and Rizwan and Khan [33] reported significant positive aftermarket returns of privatization IPOs in the long run. Thus, we expect a positive relationship between these two variables. IPO performance may differ significantly across the industries [2,19,27]. We include three industry dummies to control for the industry effect.

## Methodology

### Measures of aftermarket performance

The event–time portfolio approach method is used in this study to measure the abnormal aftermarket returns of IPO firms [14,19,34] by calculating CAARs and BHRs for 36 months following the first trading day. Initially, we calculate the daily stock returns and daily market returns. Following Allen et al. [27], the raw return for each firm, *R*_*i*_, is calculated as

$${\text{R}}_{\text{i}1}=\left[\left(P_{it}-P_{i\left(t-1\right)}\right)/\left(P_{i\left(t-1\right)}\right)\right]$$ (3)

where *P*_*it*_ is the closing price of an IPO on a particular trading day, and _*(t-1*)_ is the previous trading day. Similarly, for the market return, *R*_*mt*_, the return is calculated from the differences in the ASPI market index values for the same time interval as above on a firm basis.

Then, the market-adjusted return for stock *i* in the *t*^th^ trading day is defined as

$$ar_{it}=r_{it}-r_{mt}$$ (4)

where *r*_*it*_ is the return for stock, *i* in the *t*^th^ trading day and *r*_*mt*_ is the market return index during the corresponding day.

Following Ritter [2] the aftermarket period returns for 36 months are calculated after converting daily data into monthly data by grouping 720 days into 36 months, assuming that there are 20 trading days in each trading month. The average market-adjusted return (AAR) on a sample of *n* stocks for the *T*^*th*^ event month is the equally weighted arithmetic average of the market-adjusted returns for each trading month, which is calculated as

$$\bar{AAR_{T}}=\frac{1}{n}\;\overset{n}{\underset{i=T}{\sum }}ar_{it}$$ (5)

The CAARs from trading month 1 to trading month *T* is the summation of the AARs (*AAR*_*T*_). In particular, the *CAAR* from event month *q* to event month *s* is the summation of *AAR*_*T*_ over various intervals during the 36-month aftermarket period:

$$CAAR_{q,s}=\overset{s}{\underset{i=q}{\sum }}\bar{AAR_{T}}$$ (6)

The calculation of t-statistics for the AR_T_ series are as follows,

$$t\left(AAR_{T}\right)=ARR_{T}*\;\frac{{\sqrt{n}}_{T}}{sd_{T}}$$ (7)

where *n*_T_ is the number of firms trading in event month T, and *sd*_T_ is the cross-sectional Standard Deviation for event month T.

The conventional t-statistic (8) is used to test the statistical significance of the CAARs [2].

$$t\left(CAAR_{iT}\right)=CAAR_{iT}*\;\frac{\sqrt{n_{T}}}{\sqrt{T*var+2*\left(T-1\right)*cov}}$$ (8)

where var is the average of the cross-sectional variations over T months of the AR_*i*, *T*,_, and cov is the first-order auto-covariance of the AR_*T*_ series, which is calculated by the correlation coefficient * cross-sectional variance.

BHRs are calculated using daily returns from the beginning of the holding period until either the end of the holding period or the delisting date, whichever is earlier [1,2]. Following Ritter [2], we excluded the initial trading day from BHR calculations

$$BHR_{it}=\overset{T}{\underset{t=1}{\prod }}\left(1+r_{it}\right)-1$$ (9)

where T is the trading month, *r*_it_ is the raw return on firm *i* in the trading day *t*, and T is the trading months (1–36).

Therefore, the market adjusted BHR [34] is defined as

$$BHR_{iT}=\overset{T}{\underset{t=1}{\prod }}\left(1+r_{it}\right)-1-\overset{T}{\underset{t=1}{\prod }}\left(1+r_{mt}\right)-1$$ (10)

where T is the trading month, *r*_*it*_ is the raw return for stock *i* in the *t*^th^ trading day, and *r*_*mt*_ is the return on the market during the corresponding period.

The average BHR for the period *T*, denoted as *BHR*_*iT*_, is the arithmetic mean abnormal return on all IPOs in the sample of size *n*:

$$\bar{BHR_{t}}=\frac{1}{n}\overset{n}{\underset{i=1}{\sum }}BHR_{iT}$$ (11)

where the BHR for stock i in the t^th^ trading day, n, refers to the number of observations.

A positive value of BHR shows that IPOs outperform in the considered period, and a negative value of BHR shows that IPOs underperform in the same period.

### Empirical *m*ethodology

The sample data for this study consist of 26 years of daily observations (total 97,125) from 1991 to 2017. Daily stock prices and market returns, were collected from the CSE data bank (https://www.cse.lk/pages/listed-company/listedcompany.component.html?status=1), after paying the subscription fees for Platinum package. While firm-level data extracted from company annual reports, and the IPO prospectus of each firm. The sample is 144 IPO issues, which is more than 70% of the total of 200 IPOs, including a total of 11 delisted firms within 36 trading months from the first trading date.

We first analyze the aftermarket returns in calendar years and on an industry basis. Then, we use cross-sectional analyses to identify the determinants of IPO aftermarket performance, followed by multiple regression analyses at the final stage. The selection of explanatory variables is based on the previous studies.

The multiple regressions used are:

$$BHR_{it}=\beta_{0}+\;\beta_{1}IR+\beta_{2}lnAGE+\beta_{3}lnSIZE+\beta_{4}SENT+\beta_{5}lnVOL+\beta_{6}lnPRC+\beta_{7}MVL+\beta_{8}PRIV+\beta_{9}HOT+\beta_{10}BRD+\beta_{11}IND+\varepsilon_{it}$$ (12)

$$BHR_{it}=\beta_{0}+\beta_{1}MAAR+\beta_{2}lnAGE+\beta_{3}lnSIZE+\beta_{4}SENT+\beta_{5}lnVOL+\beta_{6}lnPRC+\beta_{7}MVL+\beta_{8}PRIV+\beta_{9}HOT+\beta_{10}BRD+\beta_{11}IND+\varepsilon_{it}$$ (13)

where the dependent variables are the *BHR*_*i*_ for 20, 60, 120, 180, 240, 480, and 720 trading days; *AGE* denotes the firm age from its legal registration; *SIZE* denotes the gross amount of IPO proceeds; *PRI* represents the issue price of an IPO in Sri Lankan Rupees; *SENT* denotes the investor sentiment; *VOL* denotes the annual volume of IPO stock listings in the CSE; *MVL* refers to the standard deviation of daily market returns for the first 30 trading days; *HOT* denotes the hot-period issues; *PRV* denotes the privatization issues; *BRD* denotes the listed board types; and *IND* indicates three dummies for the main industries. The detailed descriptions and summary of variables are shown in Table 1.

**Table 1:** Variables in the aftermarket performance study.

Variable	Symbol	Measurement	Ex. sign
Buy and hold return	BHR	See Eq 10 in the text	+/-
Raw Initial Return	IR	IR_i1_ = [(*P*_*i*1_-*P*_*i*0_) / (*P*_*io*_)]	-
Market Adjusted Abnormal Return	MAAR	MAAR_*i*1_ = {[(1+ *IR*_*i*1_) / (1 + Rm_1_)] − 1}	-
Firm Age	AGE	The natural logarithm of the firm age from its’ incorporation	+
Issue Size	SIZE	The natural logarithm of gross proceeds received from the IPO issue	+
Board Type	BRD	A dummy variable for Main Board listed firms	-
Offer Price	PRI	The natural logarithm of IPO offer price	-
Investor Sentiment	SENT	The % change of ASPI in one month before the IPO issue	-
IPO Volume	VOL	The natural logarithm of the annual volume of listing	-
Market Volatility	MVL	The standard deviation of daily market returns for the first 40 trading days after the IPO	-
Privatization Issue	PRV	A dummy variable for privatization issues	+
Hot Issue Period	HOT	A dummy variable for hot period issues	-
Issuer’s Industry	HTL PLNT BNK	Dummy variables for hotel, plant and bank Industry’ Firms	**+/-**

## Empirical results

### Aftermarket performance measured by AARs and BHRs

Table 2 shows that the AARs and CAARs are always lower than 1% for the first 36 months after the listing day. The AARs vary between -0.15% and 0.15%. The CAARs for 144 IPOs are 0.54% over 36 months after listing. Furthermore, CAARs are all negative up to the twenty-sixth trading month and subsequently show positive returns. However, the t-statistics are not statistically significant. Moreover, both BHRs and CAARs are negative up to the twelfth trading month, and after that BHRs show positive returns, whereas CAARs show positive returns at the three-year holding period only (Table 1). On a daily basis, there are many negative returns, so CAARs are lower than BHRs. BHRs are negative in the short run, and during the long run IPOs outperform them with positive BHRs. In particular, over three years, the average BHRs are 12.46% for the sample. However, skewness adjusted t-statistics are not statistically significant.

**Table 2:** Average monthly market-adjusted returns (AARs) and BHRs.

AAR and CAAR							BHR		
Trading Month	Firms	AAR (%)	t-statistic (AAR)	CAAR (%)	t-statistic (CAAR)	Period	Firms	BHR (%)	Skewness adj. t-statistic (BHR)
**1**	144	-0.1521	-0.2533	-0.1521	-0.3643	**BHR20**	144	-3.04	-1.2497
**3**	144	-0.0796	-0.1573	-0.2547	-0.3334	**BHR60**	144	-5.09	-1.3157
**6**	142	-0.0473	-0.0990	-0.3902	-0.3540	**BHR120**	144	-7.80	-1.6408
**12**	137	-0.0153	-0.0258	-0.1431	-0.0896	**BHR240**	141	-1.38	-0.2318
**24**	132	0.0230	0.0603	-0.0967	-0.0419	**BHR480**	137	2.14	0.3603
**36**	124	0.0013	0.0036	0.5417	0.1856	**BHR720**	132	12.46	1.6326

This table indicates the average monthly market-adjusted returns (AARs), and cumulative average monthly market-adjusted returns (CAARs) for the 36 trading months of IPOs. Market-adjusted buy-and-hold returns (*BHR*_*i*_) are calculated for six periods namely BHR20 to BHR720 considering 20, 60, 120, 240, 480 and 720 trading days respectively.

### Aftermarket performance categorized by initial returns

Table 3 reveals a clear relationship between the initial returns and the aftermarket returns for both the short run and the long run. *BHR20–BHR120* are negative in the short run and gradually give positive returns in the long run. Initial returns in the highest quintile (*MAAR/IR* ≥ 120) have the worse BHRs. Nevertheless, in the short run, *BHR20–BHR120* mostly appear to be negatively related to the IPO under-pricing. In contrast, in the long run, *BHR240–BHR720* perform well for the lower initial return quintiles, whereas the higher initial returns quintile always has negative BHRs. When IPOs are initially either overpriced or underpriced, aftermarket IPO returns also underperform in the short run and then perform well in the market in the long run by generating positive BHRs and a similar pattern for both *IR* and *MAAR*. The results show that there is a considerable difference when initial IPOs are overpriced and that IPOs are more outperformed/underperformed in the aftermarket performance. However, between BHRs, only *BHR720* returns have a significant difference at the 5% level.

**Table 3:** Aftermarket performance categorized by initial returns.

	Average Aftermarket performance (%)					
Initial returns (%)	BHR20	BHR60	BHR120	BHR240	BHR480	BHR720
**Panel A**						
IR < 0	-1.21	-9.42	-13.10	-9.48	15.52	53.07**
0 ≤ IR < 10	-8.72	1.50	-3.67	6.88	11.01	19.58
10≤ IR < 50	-2.20	-7.91	-9.55	4.58	1.05	3.72
50≤ IR < 120	3.08	0.38	3.57	-1.71	-11.76	-7.87
IR ≥ 120	-6.22	-9.51	-16.10	-18.18	-9.21	-15.15
**Panel B**						
MAAR < 0	-5.97	-5.52	-8.74	-3.53	9.63	21.55
0 ≤ MAAR < 10	0.62	5.24	2.40	6.18	19.30	53.40***
10≤ MAAR < 50	-3.53	-9.14	-11.74	4.42	2.14	10.62
50≤ MAAR < 120	0.21	-5.67	-5.54	-9.50	-14.79	-8.61
MAAR ≥ 120	-6.04	-6.98	-12.69	-15.39	-13.89	-22.99*
**Panel C**						
IR overpriced	-1.21	-9.42	-13.10	-9.48	15.52	53.07
IR underpriced	-3.57	-3.86	-6.28	-0.90	1.45	2.02
Negative- positive	2.36	-5.56	-6.82	-8.58	14.07	51.05**
MAAR overpriced	-5.97	-5.52	-8.74	3.53	9.63	21.55
MAAR underpriced	-2.24	-4.97	-7.54	-0.80	0.13	10.02
Negative- Positive	-3.73	-0.55	-1.20	4.33	9.50	11.53

This table shows the aftermaret performnce categorized by initial returns. Market-adjusted buy-and-hold returns (*BHR*) are calculated for six periods namely BHR20 to BHR720 considering 20, 60, 120, 240, 480 and 720 trading days respectively. IR refers to the initial returns and MAAR refers to market adjusted abnormal returns. Sample t-statistics to test the difference between categories and the overall average returns are calculated. Two-tails sample t-statistics are used to test the difference in means (assuming unequal variances). ***, **, * denote significance at the 1%, 5%, and 10% level, respectively.

### Aftermarket performance categorized by individual measures

The complete breakdown of aftermarket returns considering different measures related to aftermarket performance are shown separately in Table 4. The IPOs of firms aged 1–4 years have lower BHRs than returns of IPOs in 5–9 years in operation. The results specify that the aftermarket returns remain highest for the firms aged 10–19 years and tend to have positive returns with mature IPOs after one year. Firms aged more than 20 years have the worst performance in the short run, and this continues up to *BHR480*. Interestingly, the positive BHRs recorded by firms aged 10–19 years are significant at the 10% level. Furthermore, following Loughran et al. [1] and Rathnayake et al. [15] firms aged less than 10 years are classified as young. Young vs. old illustrates a tendency for the age to be negatively related to the BHRs, i.e., younger firms underperform for *BHR20–BHR120* and then perform well for *BHR240–BHR720*.

**Table 4:** Aftermarket performance categorized by individual measures.

Measures	Average Aftermarket performance (%)					
	*BHR20*	*BHR60*	*BHR120*	*BHR240*	*BHR480*	*BHR720*
***AGE***						
1–4	0.92	2.77	-2.76	-6.42	-4.06	6.82
5–9	-1.45	-9.94	-11.03	-15.70	0.46	2.44
10–19	-2.60	-4.17	-0.33	25.70**	25.30*	19.78*
20<	-11.52	-12.33	-20.81	-8.45	-14.93	24.08*
***Young Vs*. *Old***						
Young: *AGE* <10	-0.13	-2.86	-6.42	-10.53	-2.16	5.02
Old: *AGE* ≥10	-6.58	-7.81	-9.47	10.28	7.49	21.67
Young-Old	6.45	4.95	3.04	-20.81**	-9.65	-16.65*
***SIZE (Rs*. *Mn*.*)***						
*SIZE* < 100	5.21**	9.87**	8.79**	14.38	21.17*	39.79**
100≤ *SIZE* < 340	-11.04**	-19.55**	-22.78**	-5.18	7.60	12.87
*SIZE ≥*340	-2.79	-4.68	-8.45	-13.09	-22.59**	-14.65**
***Small vs*. *Large***						
Small: *SIZE* ≤ 200	-0.93	1.24	-0.29	11.13	15.02	32.80
Large: *SIZE* > 200	-5.22	-11.61	-15.52	-14.07	-10.93	-8.50
Small–large	4.29	12.85	15.23	25.21**	25.95**	41.30**
***BRD***						
Main	0.94	2.43	3.11	3.40	-1.88	13.28
Secondary	-8.02	-14.50	-21.43	-7.48	7.62	11.29
Main–Secondary	8.96**	16.94**	24.54**	10.88	-9.50	1.99
***PRI* (Rs.)**						
1 to 11	-0.52	-2.41	-2.69	4.19	4.69	6.56
12 to 20	-2.86	-5.68	-5.58	-9.58	-12.13	10.66
21 to 300	-5.69	-7.16	-14.93	1.25	14.33	20.82
***MVL***						
*MVL*<28	-4.45	-2.17	-0.09	7.27	-0.02	16.87
28≤ *MVL* < 64	4.22	2.98	2.99	8.30	15.54	33.36
64≤ *MVL* < 116	-2.45	-19.02**	-26.10**	-6.24	8.27	19.58
*MVL* ≥116	-9.49	-2.16	-7.98	-14.44	-14.93	-19.71**
***VOL***						
2–5	-7.45	-8.75	-13.95	-14.44	-5.30	-1.59
6–10	3.83	4.21	12.07**	19.90**	26.05**	18.11
11–13	-2.18	-7.72	-14.64	11.64	16.80	49.69***
14–15	-4.37	-6.14	-11.27	-18.22	-27.29**	-11.03
***SENT***						
Negative *SENT*	-1.31	-1.07	-6.95	-1.60	6.22	15.17
Positive *SENT*	-5.76	-11.42	-9.13	-1.04	-4.13	8.43
Negative–Positive	4.44	10.35	2.18	-0.56	10.35	6.75
***Issue type***						
Privatisation issues	8.60	13.72	21.77	26.79	10.89	10.51
Conventional issues	-6.50	-10.69	-16.59	-9.65	-0.53	13.09
Difference	15.11**	24.40***	38.36***	36.43***	11.41	-2.59
***Hot and Cold issues***						
Cold year issues	-8.20	-6.96	-9.34	-15.59	-2.42	0.28
Hot year issues	-0.84	-4.30	-7.14	4.65	4.09	17.39
Difference	-7.36**	-2.66	-2.19	-20.23**	-6.51	-17.11*

This table shows the aftermarket performnace calculatons based on the individual measures. Market-adjusted buy-and-hold returns (BHRs) are calculated for six periods, namely *BHR20* to *BHR720*, considering 20, 60, 120, 240, 480, and 720 trading days, respectively. AGE denotes the history of the firm from its incorporation and classifies issues up to Rs. 200 million as being small and those above that figure as being large; SIZE denotes the gross proceeds from the IPO and classifies up to Rs. 200 million as being small and above Rs. 200 million as being large. Rs. is Sri Lankan Rupees; BRD denotes the listed board types; PRI denotes the offer price of the IPO; MVL denotes the standard deviation of the daily ASPI for the first 40 trading days prior to the IPO issue; VOL denotes the annual volume of listings in the stock market, and IPOs are categorized into four equal groups based on the number of IPOs went to the public annually; SENT is a proxy for investor sentiment; HOT denotes the hot-period issues and cold-period issues, respectively. Sample t-statistics are used to test the difference between categories, and the overall average *BHR*s are calculated. Two-tailed sample t-statistics are used to test the difference in mean *BHR*s (assuming unequal variances). ***, **, and * denote significance at the 1%, 5%, and 10% levels, respectively.

*SIZE* reveals the aftermarket returns grouped by the size of the IPO issue, and IPOs are separated into three subgroups with nearly equivalent numbers of IPOs. Our results show that in the long run, smaller issues perform better than do larger issues. Moreover, issues up to Rs. 200 million are categorized as small and those above this figure are categorized as large to investigate the size effect. The small vs. large category illustrates that small issues tend to outperform, except *BHR60* returns, whereas large issues underperform all over the periods. The differences in the long run, including for *BHR240* are significant at 5%.

The results show that the aftermarket returns of the Main Board listed firms were positive compared to those of the Secondary Board listed firms. *BHR480* returns show poor performance for both Main and Secondary listed boards. Conversely, the *BHR720* returns for three years show positive values. The long-term return differences between the two different boards are statistically significant up to *BHR240*, whereas the *BHR480* and *BHR720* return differences are not significant. Table 4 shows that two subgroups where the IPO shares were priced either lower than or equal to Rs. 20 performed poorly in the long run.

We divided the sample into equal four subgroups with an equivalent number of observations of 36 firms in each subgroup based on *MVL* values. When the *MVL* is high (*MVL*≥116), BHRs are always negative. The other three subgroups tend to show lower aftermarket returns in the short run, and the returns increase gradually with the passage of trading time and end up being positive. Moreover, the results show that 28≤ *MVL* < 64 subgroup records outperformed stocks continuously throughout the three years, even though values were insignificant. *VOL* indicates the four equal-sized subgroups grounded on the number of IPOs that went to the public annually. The level of underperformance remains highest for the 14–15 issues that are significant at 5% and tends to decrease when the IPO volume increases. BHRs are positive when the volume is between 6–10, whereas the returns of the other three subgroups do not show a clear pattern.

Furthermore, in the short run, IPOs underperform in both negative *SENT* and positive *SENT* in the market condition. BHRs perform worse in the positive *SENT* than in the negative *SENT*. During *BHR480* and *BHR720*, performance shows positive returns for IPOs issued at the time of negative *SENT* and returns show an increasing trend over the long-term for the negative *SENT* category. Even though the differences between mean returns in the two groups are statistically insignificant, the findings reveal a negative relationship between positive *SENT* and long-term IPO performance. Privatization issues are likely to perform better than conventional issues in the long run, up to two years. Privatized IPO issues show a trend of gradually increasing performance during the short time horizon and produce maximum returns during the first trading year of stocks. Conversely, conventional issues performing worse during the first year of trading and stars showing positive returns after the second year. The differences in the *BHR20–BHR240* during the first trading year after the IPO issue are statistically significant at the 5% level.

Furthermore, following Rathnayake et al. [15], Table 4 shows that the BHRs are segmented by hot and cold year issues. According to the results, hot issue period IPOs perform better in the long run than do cold year IPO issues. Over the short-term, both hot issue and cold issue IPOs show negative abnormal returns, with hot issues still performing better than do cold issues. The difference between the two is significant at the 5% level in the first trading month. Long-term hot issues perform well and generate positive abnormal returns throughout *BHR240–BHR720*, with a positive trend of increasing returns over longer periods.

### IPO performance categorized by industry

The plantation industry has the highest returns *BHR20–BHR120* in the short run, and those returns are significantly different from the overall average at the 5% level (Table 5). Interestingly throughout the three years, the plantation industry is the only industry that performs well and generates positive BHRs continuously. Health care, power and energy, services, and trading sector IPOs always underperform in the long run. The underperformance of the power and energy industry differs sharply from the average returns of the sample, and the difference is significant at the 1% level for less than twelve trading months. Interestingly, four industries the beverage, food and tobacco sector, the footwear and textiles sector, the hotels and travel sector, and the manufacturing sector show a similar tendency of BHRs that underperform in the short run and outperform in the long run.

**Table 5:** IPO performance by industry.

	Industry	No. of Firms	Average Aftermarket performance (%)					
			*BHR20*	*BHR60*	*BHR120*	*BHR240*	*BHR480*	*BHR720*
1	Banks, Finance and Insurance	35	-1.67	-4.46	-9.40	-12.22	-6.63	14.05
2	Beverage, Food and Tobacco	11	-3.31	-1.31	-3.81	10.78	0.11	12.63
3	Diversified Holdings	8	4.87	-1.31	-11.31	-28.78	-29.99	-41.10
4	Footwear and Textiles	4	-16.01	-12.37	-38.29	130.19***	139.01***	107.83**
5	Health Care	5	-2.16	-15.21	-24.77	-51.75*	-26.12	-11.48
6	Hotels and Travel	18	-12.49	-9.58	-0.17	12.87	28.09	55.51**
7	Information Technology	4	-8.25	-1.40	2.61	35.66	-3.73	-57.09
8	Land and Property	3	-6.24	-13.45	-23.62	-15.51	-14.85	42.25
9	Manufacturing	21	-2.05	-19.55	-25.02	-5.41	16.40	17.75
10	Motors	1	7.52	37.94	30.84	18.95	-17.65	-20.06
11	Plantation	18	11.39**	24.77**	28.60**	13.05	7.10	17.69
12	Power and Energy	8	-4.33***	-11.25***	-12.55***	-27.38	-32.64	-33.69
13	Services	2	-11.58	-14.30	-33.48	-23.03	-17.62	-28.92
14	Trading	6	-23.72*	-27.17	-28.99	-24.98	-47.35	-9.72
	Total	144	-3.04	-5.09	-7.80	-1.38	2.14	12.46

This table gives the sample distribution by the industry; the number of firms and the average aftermarket returns. Market-adjusted buy-and-hold returns *(BHR*_*i*_*)* are calculated for six periods namely *BHR20* to *BHR720* considering 20, 60, 120, 240, 480 and 720 trading days respectively. Two-tails sample t-statistics are used to test the difference in the average *BHR*s in each industry and the overall average *BHR*s in the sample (assuming unequal variances). ***, **, * denote significance at the 1%, 5%, and 10% level, respectively.

### Multiple regression analysis

First, the OLS assumptions are tested before running the multiple regressions. All the non-dummy variables are normally distributed (Table 6). All the non-dummy variables are stationary at the level according to the Augmented Dickey-Fuller (ADF) unit root test results, which are given in Table 7. As illustrated in the correlation matrix (Table 8), independent variables do not appear to be substitutes of each other since the correlation between variables is less than 0.5. Only IR and MAAR are 94% positively correlated, but we do not consider IR and MAAR in the same regression model.

**Table 6:** Shapiro-wilk normality test.

Variable	Statsitic	DF	Significanse
*BHR01*	0.917	144	0.035
*BHR03*	0.944	144	0.044
*BHR06*	0.764	144	0.000
*BHR12*	0.843	144	0.012
*BHR24*	0.758	144	0.000
*BHR36*	0.867	144	0.019
*IR*	0.755	144	0.000
*MAAR*	0.741	144	0.000
*AGE*	0.875	144	0.027
*SIZE*	0.727	144	0.000
*SENT*	0.989	144	0.044
*VOL*	0.749	144	0.000
*PRC*	0.888	144	0.022
*MVL*	0.861	144	0.025

Note: Shapiro-Wilk Normality test statistic values are recorded in the table. Market-adjusted buy-and-hold returns *(BHR*_*i*_*)* are calculated for six periods namely *BHR20* to *BHR720* considering 20, 60, 120, 240, 480 and 720 trading days respectively. IR denotes the initial returns; MAAR denotes the market adjusted abnormal return; AGE denotes the history of the firm from its incorporation; SIZE denotes the gross proceeds from the IPO; PRC denotes the issue price of an IPO in Sri Lankan Rupees; SENT is a proxy for investor sentiment; VOL denotes the annual volume of listings in the stock market; MVL refers to the standard deviation of daily market returns for the first 30 trading days after the IPO; HOT denotes the hot-period issues; PRIV denotes the privatization issues; BRD denotes the listed board types; and HTL, PLNT, and BNK are three dummies for the hotel, plantation, and banking industries, respectively. ***, **, * denote significance at the 1%, 5%, and 10% level, respectively.

**Table 7:** ADF unit root test results.

Variable	Intercept	Trend and Intercept	
*BHR01*	-11.63***	-11.93***	Level
*BHR03*	-10.14***	-10.41***	Level
*BHR06*	-10.56***	-10.69***	Level
*BHR12*	-9.79***	-9.91***	Level
*BHR24*	-11.31***	-11.28***	Level
*BHR36*	-10.64***	-10.59***	Level
*IR*	-9.29***	-9.73***	Level
*MAAR*	-9.81***	-10.23***	Level
*AGE*	-10.31***	-10.29***	Level
*SIZE*	-6.01***	-10.92***	Level
*SENT*	-11.47***	-11.67***	Level
*VOL*	-2.98**	-3.54**	Level
*PRC*	-9.29***	-10.99***	Level
*MVL*	-12.29***	-12.33***	Level

Note: Augmented Dickey-Fuller test statistic values are recorded in the table. Market-adjusted buy-and-hold returns *(BHR*_*i*_*)* are calculated for six periods namely *BHR20* to *BHR720* considering 20, 60, 120, 240, 480 and 720 trading days respectively. IR denotes the initial returns; MAAR denotes the market adjusted abnormal return; AGE denotes the history of the firm from its incorporation; SIZE denotes the gross proceeds from the IPO; PRC denotes the issue price of an IPO in Sri Lankan Rupees; SENT is a proxy for investor sentiment; VOL denotes the annual volume of listings in the stock market; MVL refers to the standard deviation of daily market returns for the first 30 trading days after the IPO; HOT denotes the hot-period issues; PRIV denotes the privatization issues; BRD denotes the listed board types; and HTL, PLNT, and BNK are three dummies for the hotel, plantation, and banking industries, respectively. ***, **, * denote significance at the 1%, 5%, and 10% level, respectively.

**Table 8:** Pearson correlation matrix.

Variables	*MAAR*	*IR*	*AGE*	*SIZE*	*SENT*	*VOL*	*PRC*	*RISK*	*PRIV*	*HOT*	*BORD*	*BNK*	*PLNT*	*HOTL*
*MAAR*	1													
*IR*	0.94***	1												
*AGE*	0.01	0.03	1											
*SIZE*	-0.29***	-0.26***	0.14	1										
*SENT*	0.24***	0.27***	0.08	-0.14	1									
*VOL*	0.02	-0.01	0.03	-0.04	-0.09	1								
*PRC*	-0.07	-0.07	0.19*	0.20*	0.09	0.32*	1							
*MVL*	-0.12	-0.09	0.11	0.23***	-0.01	-0.09	-0.09	1						
*PRIV*	0.21*	0.19*	-0.07	-0.22***	0.05	0.05	-0.06	-0.31***	1					
*HOT*	0.15**	0.14**	0.06	0.01	0.01	-0.02	-0.09	0.03	0.14**	1				
*BRD*	0.09	0.10	0.01	0.11	-0.05	-0.01	0.21**	-0.22*	0.32***	0.08	1			
*BNK*	0.02	0.07	0.07	0.15**	-0.08	-0.11	0.07	-0.01	-0.19**	-0.10	0.07	1		
*PLNT*	0.28***	0.17**	-0.12	-0.37***	0.05	-0.08	-0.13	-0.07	0.57***	0.19**	0.15*	-0.20**	1	
*HTL*	-0.12	-0.09	-0.21**	-0.12	0.08	0.02	0.02	0.04	-0.16*	-0.07	-0.08	-0.21**	-0.14*	1

Note: This table presents the Pearson correlation coefficients for the variables considered in the study. IR denotes the initial returns; MAAR denotes the market adjusted abnormal return; AGE denotes the history of the firm from its incorporation; SIZE denotes the gross proceeds from the IPO; PRC denotes the issue price of an IPO in Sri Lankan Rupees; SENT is a proxy for investor sentiment; VOL denotes the annual volume of listings in the stock market; MVL refers to the standard deviation of daily market returns for the first 30 trading days after the IPO; HOT denotes the hot-period issues; PRIV denotes the privatization issues; BRD denotes the listed board types; and HTL, PLNT, and BNK are three dummies for the hotel, plantation, and banking industries, respectively. ***, **, * denote significance at the 1%, 5%, and 10% level, respectively.

Table 9 shows OLS results for the aftermarket returns of six dependent variables, *BHR20–BHR720*. We used Eqs 12 and 13 for each BHR, considering *IR* and *MAAR*, respectively. The multiple regression models explain approximately between 10%–22% of the overall variations of IPO aftermarket performance in the considered sample, which is measured by R^2^. According to our results, the *BHR20*, *BHR120*, *BHR240*, and *BHR720* regression models have significant F-statistic values.

**Table 9:** Multiple regression analysis.

Variables	Average Aftermarket performance (%)											
	*BHR20*		*BHR60*		*BHR120*		*BHR240*		*BHR480*		*BHR720*	
	Eq (12)	Eq (13)	Eq (12)	Eq (13)	Eq (12)	Eq (13)	Eq (12)	Eq (13)	Eq (12)	Eq (13)	Eq (12)	Eq (13)
*IR/ MAAR*	-0.023	-0.046	-0.042	-0.080	-0.079*	-0.137**	-0.119**	-0.203**	-0.121**	-0.202**	-0.197***	-0.333***
*AGE*	-0.009	-0.009	-0.003	-0.003	0.033	0.033	0.089	0.088	0.040	0.039	0.141*	0.140**
*SIZE*	-0.009	-0.008	-0.009	-0.011	-0.001	-0.002	-0.060	-0.063	-0.109*	-0.111**	-0.116*	-0.120**
*SENT*	0.028	0.029	0.036	0.038	0.059	0.060	0.052	0.054	0.034	0.034	0.023	0.024
*VOL*	0.059	0.061	0.098	0.101	0.133	0.139	0.142	0.151	0.029	0.037	0.198	0.212
*PRI*	-0.059**	-0.059**	-0.068**	-0.068**	-0.119**	-0.119**	-0.058	-0.057	0.046	0.047	0.040	0.042
*MVL*	-0.002**	-0.001**	-0.001	-0.001	-0.001	-0.001	-0.001	-0.001	-0.001	-0.001	-0.002**	-0.002***
*PRV*	0.039	0.040	0.130	0.131	0.275**	0.274**	0.479***	0.481***	0.168	0.169	-0.180	-0.178
*HOT*	-0.074	-0.072	-0.116	-0.113	-0.088	-0.084	-0.018	-0.015	0.043	0.046	0.171	0.176
*BRD*	0.069	0.069	0.173*	0.172*	0.231**	0.229**	0.087	0.083	-0.100	-0.104	-0.004	-0.014
*BANK*	0.043	0.045	0.101	0.103	0.147	0.147	-0.007	-0.005	-0.104	-0.103	-0.041	-0.043
*PLNT*	0.135	0.150	0.255	0.281	0.274	0.318	-0.250	-0.187	-0.193	-0.131	-0.164	-0.267
*HTL*	0.077	0.082	0.013	0.005	0.221	0.209	0.207	0.192	0.195	0.180	0.543**	0.524**
*C*	-0.092	-0.062	0.008	0.051	-0.282	-0.238	0.752	0.810	1.965*	2.015*	1.653	1.746
R^2^	0.155	0.162	0.119	0.126	0.167	0.176	0.147	0.162	0.109	0.119	0.195	0.215
Prob(F-stat)	0.347	0.031**	0.189	0.150	0.024**	0.015**	0.071*	0.038**	0.320	0.234	0.013**	0.005***
Observations	144	144	144	144	144	144	141	141	137	137	132	132

This table shows the regression results. Market-adjusted buy-and-hold returns *(BHR*_*i*_*)* are calculated for six periods namely *BHR20* to *BHR720* considering 20, 60, 120, 240, 480 and 720 trading days respectively. IR denotes the initial returns; MAAR denotes the market adjusted abnormal return; AGE denotes the history of the firm from its incorporation; SIZE denotes the gross proceeds from the IPO; PRC denotes the issue price of an IPO in Sri Lankan Rupees; SENT is a proxy for investor sentiment; VOL denotes the annual volume of listings in the stock market; MVL refers to the standard deviation of daily market returns for the first 30 trading days after the IPO; HOT denotes the hot-period issues; PRIV denotes the privatization issues; BRD denotes the listed board types; and HTL, PLNT, and BNK are three dummies for the hotel, plantation, and banking industries, respectively. ***, **, * denote significance at the 1%, 5%, and 10% level, respectively.

*IR* and *MAAR* have a negative relationship with *BHR20–BHR720* throughout all the periods. Even the short-term relationship is insignificant, and in the long run there is a significant relationship with BHRs. Our results are in line with the divergence of opinion hypothesis [2,10,13]. In the short run, the *lnAGE* coefficient has a negative sign, and it is statistically insignificant. For the *BHR720* period, age and aftermarket returns have a significant positive relationship, which contradicts the previous findings [2,17,35] and the fundamentals of risk–return theory. The coefficient of the *lnSIZE* has a negative relationship with *BHRs*, and in the long run, including the *BHR480* and *BHR720* relationship, is significant at the 5% level, as supported by several studies [17,27].

The signs of the two *BRD* and *lnPRI* variables are not constant during the sample periods. Although the estimated coefficient on *BRD* has a positive sign in the short run, it is statistically significant at *BHR60* and *BHR120* aftermarket returns. *BRD* has an insignificant negative relationship with *BHRs* in the long run. *lnPRI* shows a significant negative relationship with *BHR*s in the short run and a positive relationship in the long run. *MVL* coefficient values are always negative and very low. Interestingly, *BHR20* and *BHR720* coefficients for *MVL* are statistically significant, thus supporting the hypothesis and previous studies [6,17,25]. Further, Wald test results indicate that five coefficients of ex-ante uncertainty are simultaneously equal to zero in all the models, and the results are not supported by the ex-ante uncertainty hypothesis. OLS results show an insignificant positive relationship between *lnVOL* and *BHR20–BHR720* throughout the all periods, which is similar to the findings of Allen et al. [27] and Hensler et al. [28]. Also, *BHR20–BHR720* are positively related with *SENT* across the all regression models, which is not consistent with the investor sentiment hypothesis. However, values are not statistically significant.

Consistent with previous studies [32,33], *PRV* record positive signs of the coefficients for the BHRs except for *BHR720* returns, and the coefficient values are significant for *BHR120* and *BHR240* at the 5% level. The *HOT* dummy variable coefficients are negative in the short run, and the long-time horizon coefficient values are positive. Regression results indicate that *PLNT*, *HTL*, and *BNK* industries have a positive, though not statistically significant, relationship with short-term aftermarket returns. Over the longer time horizon, *HTL* coefficients are still positive, and the other two industry coefficients turn negative. For the *HTL* sector, the only coefficient of *HTL* is significant at the 5% level for *BHR720* returns. Nevertheless, we used the Wald test to test for the joint hypothesis for industry effect (Table 10) and found that the three coefficients of industries are simultaneously equal to zero.

**Table 10:** Wald test results.

	Average Aftermarket performance (%)					
	*BHR01*	*BHR03*	*BHR06*	*BHR12*	*BHR24*	*BHR36*
**Ex-ante uncertainty**						
*Eq (12) IR*	1.352 (0.254)	1.145 (0.338)	1.787 (0.135)	1.193 (0.317)	1.271 (0.285)	1.614 (0.175)
*Eq (13) MAAR*	1.371 (0.247)	1.168 (0.328)	1.787 (0.135)	1.238 (0.293)	1.340 (0.259)	1.708 (0.153)
**Industry Effect**						
*Eq (12) IR*	1.446 (0.232)	1.036 (0.379)	1.354 (0.259)	0.993 (0.398)	0.777 (0.509)	1.426 (0.239)
*Eq (13 MAAR*	1.663 (0.178)	1.203 (0.311)	1.504 (0.217)	0.706 (0.551)	0.597 (0.618)	1.447 (0.233)

Note: This table presents the Wald joint hypothesis test results. Market-adjusted buy-and-hold returns (BHRi) are calculated for six periods namely BHR20 to *BHR720* considering 20, 60, 120, 240, 480 and 720 trading days respectively. Chi-square test statistics values are given in the table, and the probability of chi-squared values are recorded in parenthesis. ***, **, * denote significance at the 1%, 5%, and 10% level, respectively.

In the final stage of multiple regression analysis, we checked for the heteroscedasticity and autocorrelation errors in the results (Table 11). Using the Breusch–Pagan, autoregressive conditional heteroscedasticity, and White’s heteroskedasticity tests, we obtained similar results showing that the model residuals do not consist of heteroscedasticity errors. Also, we conducted two autocorrelation tests, the Breusch–Godfrey and Durbin–Watson tests, and ensured that our multiple regression results were free from autocorrelation errors.

**Table 11:** Heteroscedasticity & autocorrelation tests.

	Average Aftermarket performance (%)					
	*BHR01*	*BHR03*	*BHR06*	*BHR12*	*BHR24*	*BHR36*
**Panel A**						
**Breusch-Pagan / Cook-Weisberg Heteroscedasticity test**						
*Eq (12) IR*	15.612 (0.275)	12.061 (0.523)	14.473 (0.341)	14.041 (0.331)	14.033 (0.371)	20.189 (0.191)
*Eq (13) MAAR*	17.218 (0.189)	12.487 (0.488)	15.322 (0.287)	14.801 (0.319)	14.164 (0.362)	19.561 (0.107)
**ARCH Heteroscedasticity Test**						
*Eq (12) IR*	2.294 (0.129)	0.0635 (0.801)	0.001 (0.976)	0.113 (0.736)	0.324 (0.569)	0.035 (0.851)
*Eq (13) MAAR*	2.056 (0.152)	0.062 (0.803)	0.000 (0.992)	0.133 (0.715)	0.371 (0.542)	0.132 (0.716)
**White test**						
*Eq (12) IR*	0.345	0.731	0.655	0.861	0.999	0.938
*Eq (13) MAAR*	0.180	0.796	0.837	0.819	0.999	0.850
**Panel B**						
**Breusch-Godfrey LM autocorrelation test**						
*Eq (12) IR*	0.975 (0.324)	0.239 (0.624)	1.282 (0.257)	2.208 (0.137)	0.271 (0.603)	0.061 (0.804)
*Eq (13) MAAR*	1.101 (0.294)	0.287 (0.592)	1.351 (0.245)	1.946 (0.163)	0.415 (0.519)	0.008 (0.993)
**Durbin Watson d autocorrelation test**						
*Eq (12) IR*	2.146	1.922	1.829	1.768	2.132	1.883
*Eq (13) MAAR*	2.156	1.916	1.826	1.787	2.151	1.921
d_L_	1.550	1.550	1.550	1.550	1.550	1.472
d_U_	1.924	1.924	1.924	1.924	1.924	1.949
Decision	no	indecision	no	indecision	no	indecision

Note: This table presents the heteroscedasticity and autocorrelation test results. Decision rule: dL < t statistic > dU = Zone of indecision, t statistic > dU = No autocorrelation, t statistic < dU = Positive autocorrelation. Market-adjusted buy-and-hold returns (BHRi) are calculated for six periods namely BHR20 to *BHR720* considering 20, 60, 120, 240, 480 and 720 trading days respectively. Chi-square test statistics values are given in the table, and the probability of chi-squared values are recorded in parenthesis. ***, **, * denote significance at the 1%, 5%, and 10% level, respectively.

### Robustness check

For the robustness check, we repeated our multiple regression analysis by removing 11 delisted firms which occurs during the 720 trading days from the IPO issue. Our overall results regarding the aftermarket performance of IPOs still hold, but there are very few changes (Table 12). We have found the signs of all explanatory variables to be almost identical and unchanged from the results in Table 9, except for two minor cases. First, the *HOT* coefficients are positive in all of *BHR20–BHR720* in the new regression results. Second, *HTL* sector IPOs show a negative relationship in the *BHR20* and *BHR60* periods and later all show positive aftermarket returns. However, the new results have created some variations in the significance of the variables. Interestingly, all R^2^ values are increased, and the significance of the F-statistic remains the same in the new results. Thus, we conclude that our results are robust.

**Table 12:** Robustness check- multiple regression results.

Variables	Average Aftermarket performance (%)											
	*BHR20*		*BHR60*		*BHR120*		*BHR240*		*BHR480*		*BHR720*	
	Eq (12)	Eq (13)	Eq (12)	Eq (13)	Eq (12)	Eq (13)	Eq (12)	Eq (13)	Eq (12)	Eq (13)	Eq (12)	Eq (13)
*IR/ MAAR*	-0.039*	-0.067**	-0.050*	-0.088*	-0.073*	-0.128**	-0.118**	-0.205***	-0.115*	-0.193**	-0.202**	-0.337***
*AGE*	-0.017	-0.016	0.004	0.005	0.034	0.035	0.083	0.085	0.024	0.026	0.147**	0.149**
*SIZE*	-0.009	-0.007	-0.018	-0.020	-0.001	-0.004	-0.056	-0.060	-0.090	-0.093	-0.102	-0.107
*SENT*	0.037	0.039	0.014	0.012	0.026	0.029	0.075	0.079	0.046	0.049	0.024	0.030
*VOL*	0.025	0.025	0.043	0.044	0.104	0.105	0.036	0.038	0.006	0.003	0.082	0.088
*PRI*	-0.058**	-0.058**	-0.001	-0.001	-0.058	-0.058	-0.039	-0.039	0.048	0.049	0.056	0.057
*MVL*	-0.001**	-0.001**	-0.001	-0.001	-0.001	-0.001	-0.001	-0.001	-0.001	-0.001	-0.002***	-0.002***
*PRV*	0.036	0.038	0.093	0.096	0.202	0.205	0.450***	0.455***	0.159	0.163	-0.223	-0.216
*HOT*	0.167**	0.172**	0.105	0.111	0.062	0.071	0.228	0.242	0.069	0.079	0.237	0.252
*BRD*	0.039	0.038	0.044	0.042	0.120	0.117	0.023	0.019	-0.098	-0.102	-0.049	-0.057
*BANK*	0.076	0.077	0.063	0.065	0.063	0.065	-0.031	-0.027	-0.114	-0.113	-0.045	-0.046
*PLNT*	0.189*	0.210**	0.278**	0.307**	0.290**	0.331**	-0.186	-0.120	-0.146	-0.087	-0.354	-0.456
*HTL*	-0.107	-0.109	-0.025	-0.027	0.139	0.135	0.171	0.165	0.111	0.107	0.661**	0.653**
*C*	-0.052	-0.025	0.070	0.111	-0.314	-0.258	0.762	0.854	1.685	1.751	1.544	1.660
R2	0.202	0.212	0.128	0.139	0.161	0.174	0.175	0.195	0.091	0.101	0.203	0.223
Prob(F-stat)	0.009***	0.005***	0.195	0.137	0.059*	0.033**	0.033**	0.013**	0.559	0.441	0.012**	0.005***
Observations	133	133	133	133	132	132	131	131	130	130	127	127

This table presents the robustness regression results after excluding 11 delisted firms from the sample. Market-adjusted buy-and-hold returns *(BHR*_*i*_*)* are calculated for six periods namely *BHR20* to *BHR720* considering 20, 60, 120, 240, 480 and 720 trading days respectively. IR denotes the initial returns; MAAR denotes the market adjusted abnormal return; AGE denotes the history of the firm from its incorporation; SIZE denotes the gross proceeds from the IPO; PRC denotes the issue price of an IPO in Sri Lankan Rupees; SENT is a proxy for investor sentiment; VOL denotes the annual volume of listings in the stock market; MVL refers to the standard deviation of daily market returns for the first 30 trading days after the IPO; HOT denotes the hot-period issues; PRIV denotes the privatization issues; BRD denotes the listed board types; and HTL, PLNT, and BNK are three dummies for the hotel, plantation, and banking industries, respectively. ***, **, * denote significance at the 1%, 5%, and 10% level, respectively.

## Conclusion

This study focused on the evaluation of the performance of initial price offerings (IPOs) price performance up to 36 months including the listing day in terms of market-adjusted buy and hold returns (BHRs) and market-adjusted cumulative average returns (CAARs) and the practicality determinants at the time of IPO issues to find explanations for the IPO aftermarket performance. Average market-adjusted returns and CAARs are always lower than 1%. Averagely abnormal returns are negative in the short run, and abnormal returns gradually become positive in the long run. Over the three years, IPOs outperform with positive 12.46% BHRs. We found that initial returns have a long-term significant negative relationship with all BHRs and that the outcomes are consistent with the divergence of opinion hypothesis. Market volatility and aftermarket returns are negatively related throughout the all considered periods. Privatized IPOs show a significant positive relationship with one-year aftermarket returns. Hot issue period IPOs are positively related with first trading month aftermarket returns, while other periods are not significant. Similarly, plantation sector IPOs show a positive and significant relationship in short run BHRs. We do not accept the ex-ante hypothesis in aftermarket performance as five variables age of the firm, issue size, listed board effect, market volatility, and the IPO price are jointly not significant. Aftermarket returns are positively related with investor sentiment, and the annual volume of listings are based on the firm went to the public. For the robustness check, we re-estimated the multiple regressions by using the sample of 133 firms after removing delisted companies from the original sample. We found that the signs of most of the explanatory variables are unchanged and remained the same as the full sample results.

Consequently, we suggest that investors should hold their subscriptions of IPO shares for a prolonged time frame, usually exceeding two years, as the dynamic of shares rewards the investors with positive abnormal returns in the long run. Though intrinsic characteristics of IPO firms may constitute a bias to this pattern, it is still worthwhile for investors in emerging stock exchanges to monitor the performance of IPO firms over the long-run.

## Supporting information

S1 Data (XLSX)
